# Glaucoma and its predictors among adult patients attending ophthalmic outpatient department: a hospital-based study, North West Ethiopia

**DOI:** 10.1186/s12886-021-02168-y

**Published:** 2021-11-19

**Authors:** Gashaw Mehiret Wubet, Abiyu Ayalew Assefa

**Affiliations:** 1 School of Medicine, College of Health Science, Debre Tabor University, Debre Tabor, Ethiopia; 2 Department of Public Health, Hawassa College of Health Science, P.O.Box: 84, Hawassa, Ethiopia

**Keywords:** Cross-sectional study, Ethiopia, Glaucoma, Predictors, Prevalence

## Abstract

**Background:**

Nearly 1.9% of global blindness is caused by glaucoma and this is sadly high in Africa which is around 15% and In Ethiopia, glaucoma is responsible for 5.2% of blindness. It is also the fifth cause of blindness in Ethiopia. Scarce information is available regarding glaucoma in Ethiopia; hence we determined the proportion of glaucoma and its associated factors in North West Ethiopia which will be utilized for future related researches and different stakeholders.

**Methods:**

Institution based cross-sectional study was conducted from September 1/2020 to February 30/21 among 258 adults aged 40 and above years old. The participants were selected using the systematic random sampling technique. The data was collected using an interview-administered questionnaire. Binary and Multi-variable logistic regressions were fitted to identify independent predictors of glaucoma. *P*-value less than 0.05 were used as a cutoff point for declaring statistical significance.

**Results:**

The prevalence of glaucoma was 66(26%) with a 95% CI of 17.7, 35.4%). Individuals with Positive family history of glaucoma (AOR: 3.72, 95% CI: 1.03–3.53), age (AOR: 3.21, 95% CI: 1.92–5.99) and elevated intraocular pressure (AOR: 3.09, 95% CI: 1.45–6.59) were statistically significant contributing factors for the development of glaucoma.

**Conclusion:**

The study found a relatively high proportion of glaucoma in the study area, which is primarily a disease of the elderly. Age, elevated intraocular pressure, and positive family history of glaucoma was contributing factors for the emergence of glaucoma. Therefore, establishing public awareness programs about the identified risk factors for the prevention and early detection of cases is essential.

## Background

Glaucoma is a group of ocular disorders that involves progressively damaged optic nerve and is characterized by progressive optic neuropathy resulting in a characteristic appearance of the optic disc and a specific pattern of irreversible visual field defects [1, 2].

The two most common forms of the disease are primary open-angle glaucoma (POAG) and primary angle-closure glaucoma (PACG), with different patterns of disease occurrence [3]. Secondary glaucoma can result from trauma and certain medications such as corticosteroids, inflammation, tumor, or conditions such as pigment dispersion or pseudo-exfoliation [4]. Older age, family history of glaucoma, black race, use of systemic or topical corticosteroids, and high intraocular pressure are some of the risk factors that require prompt assessment by eye care practitioners for evaluation of glaucoma [5].

People with glaucoma may show the following clinical manifestations; blurred vision, eye pain, headache, and hyperemia with or without systemic disease and medication in case of secondary glaucoma [6]. Early diagnosis of glaucoma can be challenging because there is no single perfect reference standard for establishing the diagnosis. The presence of characteristic visual field defects can confirm the diagnosis, but as much as 30 to 50% of retinal ganglion cells may be lost before defects are detectable by standard visual field testing [7].

Glaucoma can be diagnosed based on the clinical presentation of the patient, examination with Slit-lamp microscopy, tonometric measurement of IOP, evaluating the structural effect of IOP with optic disc imaging [8].

Glaucoma has been termed as the “silent thief of sight” since the loss of vision often occurs gradually over a long period, and symptoms only occur when the disease is quite advanced [9]. It is a preventable cause of blindness. Late diagnosis and inadequate treatment have been attributed as the major causes of blindness in glaucoma. Blindness in glaucoma cannot be cured, but if the disease is detected in its early stages, its progress can be arrested and in most cases, the sight can be saved. As evidence shows, late diagnosis of glaucoma is an important risk factor for subsequent blindness [10].

Glaucoma is the second leading cause of blindness after cataracts and the leading cause of irreversible blindness in the world [10, 11]. In 2013, It has been estimated that 64.3 million people were affected by glaucoma globally, increasing to 76.0 million in 2020 and 111.8 million in 2040 [12]. The global estimated bilaterally blindness from glaucoma was projected to increase from 8.4 million in 2010 to 11.1 million by 2020 [11, 12].

Tools that help to assess and diagnose glaucoma are Slit-lamp microscopy, Tonometry, Gonioscopy, Ophthalmoscopy, and Perimetry which is used to measure intraocular pressure, central corneal thickness, anterior chamber depth, lens thickness, and axial length [10, 13–15].

The World Health Organization (WHO) recommended its member countries combat the public health problem of glaucoma through a program approach. Additionally, World Glaucoma Week (WGW) is conducted every year for 1 week during March to provide more emphasis on awareness of glaucoma. To plan the strategies, it is of the highest significance that the prevalence, distribution, various subtypes in a region, and risk factors of glaucoma are identified.

Despite a large amount of time and resources spent on treatment trials, there are still few effective treatments and limited involvement to reduce the incidence of glaucoma [16]. In general, evidence-based information is lacking regarding the proportion and contributing factors of glaucoma in Ethiopia particularly in the study area despite the existence of the problem in routine health services. The study will provide baseline information for stockholders and health planners to take effective measures early before the advancement of the disease to an irreversible stage. In addition, it is important for early detection and prevention of blindness and to improve the quality of life of people with Glaucoma. This study is also important for the Ministry of Health and the local Regional Health Bureaus to draft a policy on the prevention and control of complications associated with glaucoma. Therefore, the purpose of this study was to determine the magnitude of glaucoma and its predictors among adult patients in Debre Tabor comprehensive specialized hospital.

## Methods

### Study area and period

The study was carried out in Debre Tabor Comprehensive specialized hospital from September 1/2020 to February 30/2021. Debre Tabor is located North West of the Amhara Region of Ethiopia, 103 km from Bahir Dar (Regional Capital), and 666Km from Addis Ababa (Capital city of Ethiopia). It is one of the oldest hospitals in the Amhara region, which provides services for about 5 million populations. The hospital is currently used as a referral center for district hospitals in the zone and a teaching hospital for medical and health science students of Debre Tabor University. The ophthalmic clinic is one department of the hospital with its minor and major operating units.

### Study design and population characteristics

An institution-based cross-sectional study design was implemented. All patients, aged 40 and above years old who visited the ophthalmic outpatient department of Debre Tabor Comprehensive specialized hospital were the source population, and patients aged 40 and above years old visited the ophthalmic outpatient department of Debre Tabor Comprehensive specialized hospital during the time of data collection were the study populations.

### Eligibility criteria

All patients, 40 years of age and above were eligible to participate in this study while patients who were failed to communicate because of severe illness were excluded from this study.

### Sample size determination

The sample size was determined using a single population proportion formula (*n* = Z ^2^ * p (1-P) /d^2^) for a crossectional survey based on the following assumptions: the prevalence of glaucoma (p) (9.79%) at the University of Gondar Tertiary Eye Care and Training Center, Ethiopia [17] with 95% level of confidence and 3% tolerable margin of error, the sample size required was 376. Adjustment using the correction formula was made to calculate the exact sample size since the total source population (N) was below 10,000. Therefore, the final required sample size was 279 by considering a non-response rate of 5%.

### Sampling procedure

A systematic random sampling technique was used to select the study participants. A total of 956 patients were visiting the ophthalmic outpatient department over the last six-month period. Therefore, we calculated the sampling interval (k = N/*n* = 956/279 ≈ 3). Then the lottery method was used to get the random starter number which was 2. The first individual was taken as nth and the next was by sampling fraction (k = 3). And then every 3 patients were interviewed until the allocated sample size was achieved.

### Data collection procedure

The questionnaire was developed after reviewing different kinds of literature [3, 11, 13, 17, 18]. The questionnaire consisted of demographic data, socioeconomic information, clinical and behavioral characteristics of patients. It was first prepared in English and then translated to Amharic (local language) to facilitate communication. Pretest was conducted outside the study hospital and modification was made before the actual data collection. Data collectors have obtained permission to proceed with interviewing. The data was collected by the trained two BSC nurses.

### Data quality control measure

To ensure the quality of the data every day after data collection, the questioner was reviewed for completeness, accuracy, and clarity by the data collectors.

### Data processing and analysis

The collected data was cleaned, entered, and analyzed using SPSS version 23. To explain the study population concerning relevant variables, data were described using summary measures (frequency, proportion means). Proportions estimated along with 95% CI level. Both bivariate and multivariable logistic regression analyses were carried out. Variables with a *p*-value < 0.25 in the bivariate analysis were fitted into the multivariable logistic regression model for the prediction of determinants [19]. The Hosmer-Lemeshow goodness-of-fit statistics were used to assess whether the necessary assumptions for the application of multiple logistic regression were fulfilled and it was non-significant (*p* = 0.76). Crude and adjusted odds ratio with 95% confidence interval was computed. The Adjusted Odds ratio with a 95% confidence interval is used to measure the strength of association and the actual predictors of the outcome variables. *P*-values less than 0.05 were used as a cutoff point for declaring statistically significant.

### Study variables

#### Dependent variable

Glaucoma.

#### Independent variables

socio-demographic, socioeconomic, clinical, and behavioral factors.

### Examinations

#### Measurement of Intra-Ocular-Pressure (IOP)

By using trans- palpebral tonometry: Appling topical anesthetic and dilating drops then we have instructed the patient to position their head into a device called the slit lamp. After that, a small tip gently touches the surface of the eye by using tonometry, and the eye pressure was measured. The eye pressure is measured based on the force required to gently flatten a fixed area of the cornea.

#### Visual acuity measurement

Visual acuity is a measure of the ability of the eye to distinguish the details of objects. Visual acuity testing is part of every eye examination. We measured by using the following equipment; Multi-letter Snellen chart and Plain occluder, card, or tissue.

After explaining the procedure and ensuring good natural light or illumination on the chart.

Positioned the patient, sitting or standing, six meters away from the 6-m Snellen or tumbling E chart (or 3 m away from the 3-m Snellen or E chart). Testing and recording visual acuity. Test the eyes one at a time, usually starting with the right eye, without any spectacles.

Ask the patient to cover the left eye with the plain occluder, card, or tissue.

#### Visual field measurement

We have measured the visual field in degrees from the central fixation in four quadrants: temporal (toward patient’s ear), nasal (toward patient’s nose), superior (upper, or above center), and inferior (lower, or below center).

### Glaucoma definition

In this study, the diagnosis and classification of glaucoma were based on the criteria defined by the International Society for Geographical and Epidemiological Ophthalmology (ISGEO) [20].

Cases were classified as category 3 according to the ISGEO scheme and made by: Visual acuity < 3/60 and IOP > 99.5th percentile (> 21 mmHg) OR Visual acuity < 3/60 and the eye had evidence of glaucoma filtering surgery or medical records were available confirming glaucomatous morbidity [20].

#### Primary open angle

An eye that does not have evidence of angle-closure on Gonioscopy, and where there is no identifiable secondary cause.

#### Primary closed angle

An eye with an occludable drainage angle and where there is no identifiable secondary cause.

## Results

### Socio-demographic characteristics of study participants

This study included 253 participants using a systematic sampling technique with a 90.7% response rate. The survey drew most heavily from Amhara 197 (77.5%) and followed by Oromo 26 (12.5%) ethnic groups. In this study, 150(59%) of them were male. The mean (±SD) age of the participant was found to be 63 (±12.54) years. 58 (23%) of the Amhara ethnic group had glaucoma among detected cases and by religion, orthodox had a high prevalence of glaucoma which is about 53 (20.8%). About 54 (21.3%) of married respondents had been detected as having glaucoma. Forty (15.6%) of the respondents having an income of > 48$ per month had glaucoma while 46% of respondents having an average monthly income of > 48$ had no diagnosis of having glaucoma. Concerning occupational status, 39 (15.6%) of farmers had glaucoma and 91 (30.6%) of them had no glaucoma (Table 1).

**Table 1:** Socio-demographic characteristics of study participants by glaucoma, South Gondar Zone, North West Ethiopia, 2021(*n* = 253)

Factors	Glaucoma	
	Yes n (%)	No n (%)
Age		
40–49	3 (1.2)	34 (13.5)
50–59	16 (6.3)	50 (19.8)
60–69	39 (15.4)	55 (21.7)
> = 70	8 (3.2)	48 (19.0)
Sex		
Male	48 (18.8)	111 (43.7)
Female	18 (7.3)	76 (30.0)
Religion		
Orthodox	53 (20.8)	122 (48)
Muslim	13(5.2)	53 (20.8)
Protestant	–	13 (5.2)
Marital status		
Married	54 (21.3)	147 (58)
Single	8 (3.2)	8 (3.2)
Divorced	2 (0.7)	11 (4.2)
Widowed	–	8 (3.2)
Separated	2 (0.8)	13 (5.2)
Occupation		
Farmer	39 (15.6)	91 (30.6)
Government	16 (6.2)	35 (14)
Merchant	5 (2.0)	21 (8.3)
house wife	3 (1.2)	10 (4.0)
Others^a^	3 (1)	18 (7.1)
Income per month		
< =18$	0	8 (3.2)
18–31$	0	29 (11.5)
31–48$	26 (10.4)	34 (13.5)
> 48$	40 (15.6)	117 (46.2)
Ethnicity		
Amhara	58 (22.9)	139 (55)
Oromo	5 (2.0)	21 (8.3)
Tigray	3 (1.2)	19 (7.3)
Others^b^	–	8 (3.2)
Educational level		
Cannot read &write	14 (5.3)	33 (13.0)
Can read and write	18 (7.2)	65 (25.5)
Primary school	15 (5.9)	29 (11.4)
High school	9 (3.6)	38 (15.1)
Collage& university	10 (4.0)	24 (9.5)

^a^NB:Other: daily labor, Self-employed

^b^other: Somali, Gurage, Silte

### Medical, lifestyle, and family history of respondents

Among glaucoma cases, 35(13.5%) had some form of chronic illness. Accordingly, 16(6.3%), 8(3.2%), and 8(3.2%) of patients had hypertension, diabetes mellitus, and cardiac illness respectively. Sixteen (6.2%) patients with glaucoma had also a positive family history of glaucoma. Five patients were having a refractive error with astigmatism. Twenty-six (10.3%) patients with glaucoma were smokers (Table 2).

**Table 2:** Medical, lifestyle, and family history related factors by glaucoma among adult patients attending the ophthalmic outpatient department in Debre Tabor Comprehensive and Specialized hospital, South Gondar Zone, North West Ethiopia, 2021

Factors	Category	Glaucoma			
		Yes		No	
		Frequency	Percent	Frequency	Percent
Family history of glaucoma	Yes	16	6.2	8	3.2
	No	50	19.8	179	70.8
Duration of year illness	< 6 month	20	7.8	55	21.7
	6 month to 1 year	12	4.6	46	18.2
	> 1 year	34	13.7	86	34.0
Ocular history of;	Trauma	5	2.0	5	2.0
	Surgery	3	1.2	13	5.2
	Steroid use	8	3.2	3	1.2
	refractive error	3	1.2	8	3.2
Blood pressure	< 140/90	50	19.8	166	65.6
	> = 140/90	16	6.2	21	8.3
Chronic Illness	Hypertension	16	6.2	18	7.3
	Diabetes mellitus	8	3.2	8	3.2
	Cardiac	8	3.2	3	1.2
	Chronic kidney disease	3	1.2	5	2.0
	None	31	12.3	153	60.5
Previous eye examination	Yes	105	41.5	11	4.2
	No	10	4.0	81	32.0
Types of Refraction error	Myopia	–	–	6	6.2
	Astigmatism	5	2.0	5	2.0
	None	61	24.1	167	66.0
Smoking status	Yes	26	10.3	31	12.3
	No	40	15.8	156	61.7

### Prevalence of glaucoma

**Fig. 1:**
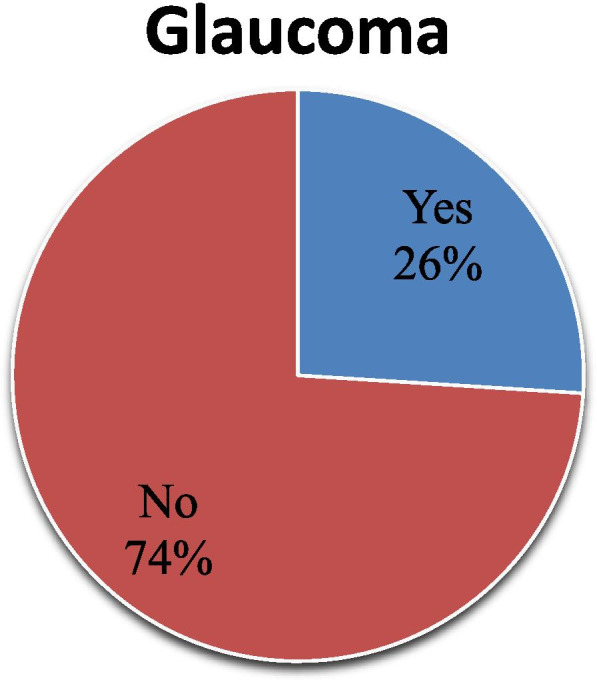
Prevalence of glaucoma among adult patients attending the ophthalmic outpatient department in Debre Tabor Comprehensive and Specialized hospital, South Gondar Zone, North West Ethiopia, 2021

The prevalence of glaucoma among adult patients attending the ophthalmic outpatient department was 26% [95% CI: 17.7, 35.4%] (Fig. 1).

### Clinical sub-types of Glaucoma

**Fig. 2:**
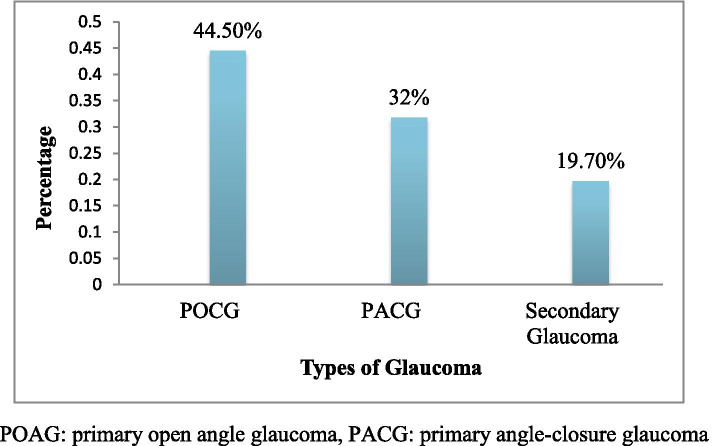
Prevalence of different types of glaucoma among adult patients attending the ophthalmic outpatient department in Debre Tabor Comprehensive and Specialized hospital, South Gondar zone, North West Ethiopia, 2021

Among the overall prevalence of glaucoma identified in the study, the most common type was primary open-angle glaucoma followed by closed-angle glaucoma which was 32(48%), and 21(32%), respectively while 13 (19.7%) of them had secondary glaucoma (Figure 2).

### Measures of intraocular pressure and visual acuity

As seen in the table below, most of the participants had normal intraocular pressure and 25% had elevated values of 21 mmHg and above, and 2.1% of them had soft eyes which are defined as IOP < 10 mmHg. Around 103(40.7%) of the participants had abnormal visual acuity, among which 61 (24%) with low vision and the rest 42 (16.7%) had been blind (NLP and CF) bilaterally. The above table also showed that most of the glaucomatous patients had elevated IOP with little difference between the right and left eyes and; there were also cases of glaucoma with normal values of IOP. Most of the glaucomatous patients had decreased visual acuity 61(24%) and 57(22%) participants in their right and left eyes, respectively (Table 3).

**Table 3:** Measure of intraocular pressure and visual acuity of glaucoma among adult patients attending the ophthalmic outpatient department in Debre Tabor Comprehensive and Specialized hospital, South Gondar Zone, North West Ethiopia. 2021(*n* = 253)

Factors		Right eye			Left eye		
		No.	Percent	Glaucoma case	No.	Percent	Glaucoma case
Intra ocular pressure	<10mmhg	5	2.1	4	5	2.1	7
	10-21mmhg	185	72.9	13	187	73.9	21
	> = 21mmhg	63	24.9	49	61	24.1	38
	Total	253	100	66	253	100	66
Visual acuity	normal(6/6–6/18)	150	59.3	5	155	61.3	9
	low vision(< 6/186/60)	61	24.1	27	56	22	27
	NLP,CF, < 3/60	42	16.7	34	42	16.7	30
	Total	253	100	66	253	100	66

Abbreviations: *NLP;* No Light Perception, *CF;* Count of Finger, *IOP;* Intraocular Pressure

### Factors contributing to Glaucoma among adult patients

In the Bi-variable logistic regression analysis, age of the patient, duration of the medical illness, intraocular pressure, presence of chronic illness, blood pressure of the patient, and family history of the patient were found to be significantly associated with glaucoma (*P* ≤ 0.25) However in multivariable logistic regression analysis only Age of the patient, duration of the illness, elevated Intraocular pressure and family history of the patient were significantly associated with glaucoma (*P* ≤ 0.05).

The age of the patient was significantly associated with glaucoma. The patient who was age greater than or equal to 60 years old was 3.2 times more likely to develop glaucoma compared to age < 60 years old [AOR:3.21,95% CI:1.92–5.99]. Elevated Intraocular pressure is also significantly associated with glaucoma. Patients whose IOP ≥21 mmHg were 3.09 times odds of having glaucoma when compared to normal IOP (< 10 mmHg), [AOR: 3.09, 95% CI: 1.45–6.59]. Patients who have a family history of glaucoma were 3.72 times more likely to develop glaucoma when compared to those who have no family history [AOR: 3.72, 95% CI: 1.03–3.53] (Table 4).

**Table 4:** Factors contributing to glaucoma among adult patients attending the ophthalmic outpatient department in Debre Tabor Comprehensive and Specialized hospital, South Gondar Zone, North West Ethiopia. 2021

Variables	Glaucoma		COR (95%CI)	AOR (95%CI)
	Yes	No		
**Age (year)**				
< 60	19	84	1	1
> =60	47	102	2.11(0.926,0.998)	3.24(1.92–5.99)*
**Chronic illness**				
Yes	35	34	4.95(3.9–16.81)	1.16(0.401–3.35)
No	32	154	1	1
**Intraocular pressure**				
< 10mmhg	4	5	1	1
10-21mmhg	13	185	0.1(3.9–16.81)	0.16(0.401–3.35)
> =21 mmHg	49	63	2.30(1.35–3.92)	3.09(1.45–6.59)*
**Duration of illness**				
< 6 month	20	55	1	1
6 month-1 year	12	46	0.72(1.21–4.67)	0.57(0.78–2.76)
> 1 years	34	86	1.51(1.53–3.69)	1.95(1.00–3.76)
**Blood pressure**				
Normal	50	166	1	1
Hypertensive	16	21	1.26(4.01–14.71)	0.59(0.58–8.19)
**Family history of glaucoma**				
Yes	16	8	6.68(1.03,-3.611)	3.72(1.03–3.53)*
No	50	167	1	1

NB. 1: reference

Abbreviations: *COR;* crude odds ratio, *AOR;* adjusted odds ratio, *CI;* confidence interval

^*^Remained significant at *P*-Value < 0.05

## Discussion

This hospital-based cross-sectional study was aimed to determine the prevalence of glaucoma and its predictors in Debre Tabor Comprehensive Specialized Hospital, North West Ethiopia. The study found 66 (26%) patients suffering from glaucoma during the study period, with 95% CI: 17.7, 35.4%) and Age of the patient, elevated Intraocular pressure and family history of the patient were remained significantly associated with glaucoma.

The prevalence of glaucoma in our study is higher compared to other studies conducted in Iran (2%) [21], Riyadh (5.6%) [22], India (2.67%) [13], Myanmar (4.9%) [23], Nepal (1%) [24], China (3.8%) [25], Thailand (3.8%) [26], Ghana (7.89%) [18], Nigeria (5.02%) [27], sub-Saharan Africa (4.5%) [11] and Gondar [17], 9.79%. The higher prevalence of glaucoma in our study might be due to sample size, nature of participants, and age of participants which tend to overestimate the magnitude. Being a referral facility of our study setting also may contribute to the increased prevalence of glaucoma.

After controlling all other variables constant old age, positive family history of glaucoma, and elevated Intraocular pressure remained significant. 

Being old age had a more likely hood to cause glaucoma by three-fold than their counterparts. This finding was similar to results in Australia [28, 29], Russia [30], Iran [21], India [13, 31], Zambia [32], Nigeria [4] and sub-Saharan Africa [11]. Supporting evidence is also shown in a study conducted in Nigeria [33], Ghana [18, 34], which reports the prevalence of glaucoma significantly increased with increasing age, from 3.7% among those aged 40 to 49 years to 14.6% among those 80 years old. Similar supporting evidence was recorded in a study conducted United Kingdom [35] which reported an increased prevalence of glaucoma with increased age. This might be due to exposure to ocular risk factors over several years of life and an age-related increase in intraocular pressures which in turn increase the chance of getting glaucoma.

This study also indicated that there is an association between a family history of glaucoma and glaucoma. Patients with a positive family history of glaucoma were 3.72 times more likely to develop glaucoma. Studies in Australia [28, 29], Sub-Saharan Africa [11], and Sudan [36] also found similar results. The reason might be due to hereditary factors. Evidence of previous study [37], also indicated linkage of several genes to adult-onset open-angle glaucoma.

In addition, intraocular pressure was associated with glaucoma in this study. Having intraocular pressure of > = 21 mmHg increases the odds of glaucoma by three times. A study in Australia [29], Singapore [38], India [13, 31], Nigeria [4], and Sub-Saharan Africa [11], also documented similar evidence. Progressive damage in the eye might occur as a result of intraocular pressure to cause glaucoma.

Finally, since this study has used a cross-sectional design, it cannot measure the temporal relationship between exposure and outcome. There is also a paucity of hospital-based studies in Ethiopia which makes it difficult to compare with the previous result.

## Conclusions

In conclusion, our study found a higher prevalence of glaucoma, which is primarily a disease of the elderly. Primary open-angle glaucoma is the most common subtype of glaucoma detected in this study. Family history of glaucoma, age, and increased intraocular pressure were significantly associated with risk factors of glaucoma in this study.

Therefore, increasing public awareness about the identified risk factors for the prevention and early detection of cases is essential. Additionally, this study calls for additional observational studies for a better understanding of factors at play.

## Data Availability

The datasets used and analyzed during the current study are available from the corresponding author on reasonable request.

## Abbreviations

AOR: Adjusted Odds Ration
CBE: Community-based Education
CI: Confidence Interval
CKD: Chronic Kidney Disease
COR: Crude Odds Ratio
DM: Diabetes Mellitus
DTCSH: Debre Tabor Comprehensive and specialized Hospital
DTU: Debre Tabor University
HTN: Hypertension
IOP: Intraocular Pressure
ISGEO: International Society for Geographical and Epidemiological Ophthalmology
LMIC: Low- and Middle-Income Countries
NTG: Normal-Tension Glaucoma
OAG: Open-Angle Glaucoma
OHT: Ocular Hypertension
PACG: Primary Angle Closure Glaucoma
POAG: Primary Open Angle Glaucoma
SPSS: Statistical Package for Social Science
